# Designing for dignity: ethics of AI surveillance in older adult care

**DOI:** 10.3389/fdgth.2025.1643238

**Published:** 2025-08-22

**Authors:** Jeena Joseph

**Affiliations:** Department of Computer Applications, Marian College Kuttikkanam Autonomous, Kuttikkanam, India

**Keywords:** artificial intelligence in eldercare, smart home technologies, surveillance ethics, human-centered design, digital privacy

## Introduction

Widespread adoption of smart home technologies (SHTs) has changed the face of eldercare. With the help of artificial intelligence, such technologies can enable older individuals to live independently with improved security. Fall sensing, sleeping pattern monitoring, and tracking behaviors have now become features in most care settings (1). Although such technologies aim to optimize care, with them come rooted ethical concerns—one such paramount one being the erosion of privacy and dignity (2, 3). As the author, I argue that technologies designed for eldercare must begin with a moral commitment to human dignity—challenging the assumption that safety and efficiency should override agency, autonomy, and emotional well-being.

Older people, already vulnerable to social isolation and intellectual decline, now face an insidious threat: continuous surveillance under the guise of care. As AI technologies guess at users’ behaviors and thought processes, the risk increases of the reduction of individuals to data points (4). What we are witnessing is not just technological advancement, but an ethical drift—one in which systems designed to care are beginning to control, and the goal of support is replaced by the logic of surveillance. This article argues that in developing AI to enable eldercare, we must move beyond the single-minded prioritization of protection and efficiency. This prompts a deeper question: What does it mean to age with dignity in a digitally mediated home? This question—not just technological, but profoundly human—is the compass for the reflections that follow.

## Dignity in the age of ambient surveillance

Dignity, difficult as it is to put into measurement, is the foundation of sound eldercare. It involves autonomy, self-respect, and the liberty to enjoy daily life without unnecessary intrusion. In the realm of AI-enhanced living, all these principles are lost (5). Though smart cameras, motion detectors, and digital aides may sense trouble sooner than human helpers, their ubiquitous presence transforms the older adult's sense of home—to no longer be sanctuaries, but surveillance zones.

It is not just about surveillance. It is about the lack of symmetry in control: data is harvested, manipulated, and frequently acted on without complete knowledge or consent from the user (6). Much older adults either have no knowledge about what data is being harvested or lack the capabilities to adjust their settings as a consequence of cognitive or technical constraints (7). This generates an ethical conflict in which security is valued over agency.

In addition, surveillance is psychologically harmful in the way it is likely to be experienced. Research has revealed that ongoing monitoring-even aimed at protection-actually produces feelings of anxiety, helplessness, or withdrawal from ordinary activities (8). The systems designed to add to quality of life might in fact decrease it in some instances.

Beyond issues of surveillance and autonomy, AI systems in eldercare also pose distinct technical and ethical risks. Algorithmic bias, for instance, can lead to unfair treatment when models are trained on non-representative datasets—more harmful in heterogeneous aging populations. Black-box decision-making makes the issue worse, as the argument in favor of system-driven intervention is difficult to grasp or dispute. In high-risk applications such as fall detection or behavioral research, the lack of explainability will only lower confidence and hasten undesirable results. In addition to substantially helping in eldercare, accuracy and speed must also be transparent, interpretable, and responsible.

## Real-world examples: a mixed record

To ground such concerns in daily life, let us turn to two real cases that bring to life the ethical challenges inherent in the use of surveillance in AI eldercare. In a pilot study in California, a fall-detection system was turned off in the home of an older adult woman after frequent false alarms triggered unannounced social service visits (9). Meant for protection, the system made her feel surveilled and powerless. Meant to support, instead, it violated her sense of agency and privacy—inseparable from the need for systems that are not only accurate but also respectful and responsive (10).

In another instance, a dementia care facility deployed emotion-recognition technology to detect signs of agitation (11). While the system occasionally flagged concerning behaviors, it also generated numerous false positives. Over time, staff began to rely more on the system's cues than on their own interactions with patients (12). This shift undermined the relational, human-centered care essential for those with cognitive decline.

These cases illustrate a critical point: when AI tools override human judgment or fail to account for users' comfort and consent, they risk turning care into control. Technologies must enhance, not replace, human connection—and must be designed with adaptability, transparency, and dignity at their core.

## A framework for dignity-first AI design

To address these challenges, I propose a “Dignity-First” framework that integrates ethical principles directly into the design and deployment of AI-based eldercare systems.

• Informed and Ongoing Consent: Because current models of consent consistently fail, particularly in the long term with cognitively vulnerable user communities—a consent system must be regarded as an ongoing, dynamic process. In this way, user dignity and autonomy are maintained through regular check-in points and user-friendly settings that enable the user or caregiver to modify permission. With the inclusion of simple, easy-to-visualize dashboards and plain-language explanations of how the data will be collected and used, such systems empower the user rather than overwhelming them.
• Data Minimization and Purpose Limitation: Collecting only the data strictly necessary for specific health objectives not only protects privacy but also reduces cybersecurity vulnerabilities. Systems that avoid storing peripheral data—such as ambient audio or location history—unless clearly justified, demonstrate ethical restraint and reduce the risk of misuse, promoting trust and responsible innovation.
• User-Configurable Privacy Settings: Recognizing that privacy needs vary from person to person, systems should provide users with the ability to set granular preferences. Features like “privacy zones” (e.g., in bathrooms) and the ability to schedule surveillance pauses ensure that users retain control. Even those with cognitive challenges should be offered simplified settings that allow them to maintain a sense of agency and comfort within their living space.
• Transparent Feedback Loops: When users lack insight into how their data is being used, it erodes trust. By integrating feedback tools—such as daily data summaries, alerts for external access, or clear explanations for triggered interventions—systems can enhance transparency. This, in turn, helps users feel more in control and more secure, reinforcing dignity through clarity.
• Design for Dignified Defaults: Since most users do not adjust factory settings, the initial configuration of a system carries significant ethical weight. By implementing privacy-protective defaults—such as limited video surveillance, local data storage, and minimal data collection—developers ensure that dignity is preserved from the start. This approach guards against intrusive overreach and prioritizes the well-being of the user without requiring them to navigate complex settings.

## The role of policy and regulation

Design alone cannot bear the burden of dignity; policymakers have a critical role in enforcing ethical standards. Just as healthcare devices are regulated for safety and efficacy, AI systems in eldercare must undergo rigorous audits to ensure ethical compliance (13). Regulatory bodies should mandate periodic privacy and usability audits, establish certification frameworks for systems that meet “dignity-by-design” criteria, and promote incentives for co-designed technologies that actively involve older adults in the development process (14). In parallel, insurance companies and public health systems can contribute meaningfully by subsidizing technologies that uphold both care quality and the fundamental rights of older adult users, ensuring that digital innovations support—not compromise—their autonomy and well-being (15).

## Co-design: nothing about us without us

One of the most powerful ways to ensure dignity is to involve older adults directly in the design process. Co-design workshops, usability testing, and participatory prototyping allow for insights that top-down engineering approaches miss. These processes must also consider diversity within aging populations, including differences in culture, language, disability, and digital literacy (16, 17).

Digital inclusion must also account for the wide range of digital literacy levels among older adults. While some may comfortably engage with apps and smart interfaces, many still rely on paper-based routines or struggle with touchscreen navigation and abstract digital icons. To ensure true usability, co-design processes should include older adults with varying levels of technological proficiency. This may involve developing hybrid solutions that combine digital and analog formats, or simplifying interfaces without sacrificing dignity or autonomy. By embracing low-tech-compatible and culturally responsive design options, we can create systems that are not only ethical but genuinely inclusive.

By treating older adults as active collaborators rather than passive recipients, designers can develop technologies that feel like companions rather than watchdogs.

## From smart homes to ethical homes

The smart homes of the future need to be turned into ethical homes—homes where care, autonomy, and privacy can coexist. This is not solely a technical challenge, but a moral imperative. Aging is not a problem to be solved, but a natural phase of life that warrants respect. Therefore, technologies should be reflective of the values we have towards the people we care about. That is about creating interfaces that enable rather than overwhelm, policy responses that safeguard without condescending, as well as developing AI systems to gain trust as opposed to commanding it.

## Conclusion

The future of eldercare is undeniably digital. But as we invite AI into the homes and lives of older adults, we must ensure that safety does not come at the cost of dignity. The right to age with privacy, agency, and self-respect must be baked into the very code of our systems. Designing for dignity is not a barrier to innovation—it is the standard we must meet to ensure technology serves humanity, not the other way around. As stakeholders—designers, policymakers, healthcare providers, and family members—we have a shared responsibility to ensure that the benefits of AI in eldercare do not overshadow the ethical need to protect what makes us human. Aging with dignity is not a luxury. It is a right.
